# Appendiceal-sigmoid fistula presenting in a man with ulcerative colitis: a case report

**DOI:** 10.1186/1752-1947-4-229

**Published:** 2010-07-29

**Authors:** Marco Santangelo, Raffaele Lanteri, Maria D'Angelo, Santo A Carnazzo, Agostino Ragalbuto, Vincenzo Minutolo, Antonio Licata

**Affiliations:** 1 Department of Surgical Sciences, Organ Transplantations and Advanced Technologies, University of Catania, Via Santa Sofia, 95123 Catania, Italy

## Abstract

**Introduction:**

Ulcerative colitis is a chronic disease characterized by diffuse mucosal inflammation limited to the colon. It mostly affects young adults, yet a large number of middle-aged and older patients with ulcerative colitis have also been reported.

**Case presentation:**

A 58-year-old Caucasian man presented to our hospital in August 2006 with continuous and diffuse abdominal pain, meteorism, fever and bloody diarrhea. He had a two-year history of ulcerative colitis. Our patient was treated with intravenous medical therapy. As his condition worsened, he underwent surgery. An explorative laparotomy revealed that the entire colon was distended and pus was found around an appendiceal-sigmoid fistula.

**Conclusions:**

Therapy for ulcerative colitis is a rapidly evolving field, with many new biological agents under investigation that are likely to change therapeutic strategies radically in the next decade. Indications for surgery are intractability (49%), stricture, dysplasia, toxic colitis, hemorrhage and perforation. To the best of our knowledge, this is the first case of an appendiceal-sigmoid fistula in a patient affected by ulcerative colitis reported in the literature. Fistulae between the appendix and the sigmoid tract are rarely reported in cases of diverticular disease and appendicitis.

## Introduction

Ulcerative colitis (UC) is a chronic disease characterized by diffuse mucosal inflammation limited to the colon. It mainly affects young adults, although a large number of middle-aged or older patients with UC have also been reported [1,2].

A hospital in the United Kingdom serving a population of 300,000 would typically see 45 to 90 new cases of inflammatory bowel disease (IBD) each year [3,4]. Indications for surgery are intractability, stricture, dysplasia, toxic colitis, hemorrhage and perforation [2].

We present a case of an unusual complication of UC, an appendiceal-sigmoid fistula complicated by UC. To the best of our knowledge, this is the first such case reported in the literature.

## Case presentation

A 58-year-old Caucasian man presented to our Surgical Unit, in August 2006 with continuous and diffuse abdominal pain, meteorism, fever (>39°C) and bloody diarrhea. Our patient had a two-year history of UC, which had been treated with oral methylprednisone and mesalazine, and with beclometasone enemas. Blood tests had revealed leukocytosis.

**Figure 1:**
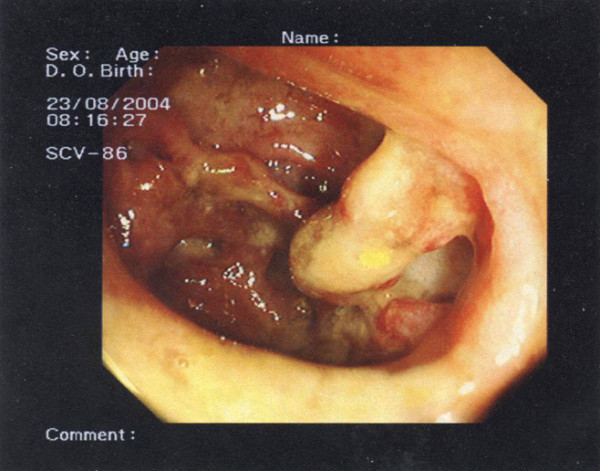
**Colonoscopy demonstrating a toxic megacolon and an appendiceal-sigmoid fistula**.

Plain abdominal radiography, colonoscopy (Figure 1) and a computed tomography (CT) scan were performed, which revealed a toxic megacolon and an appendiceal-sigmoid fistula.

**Figure 2:**
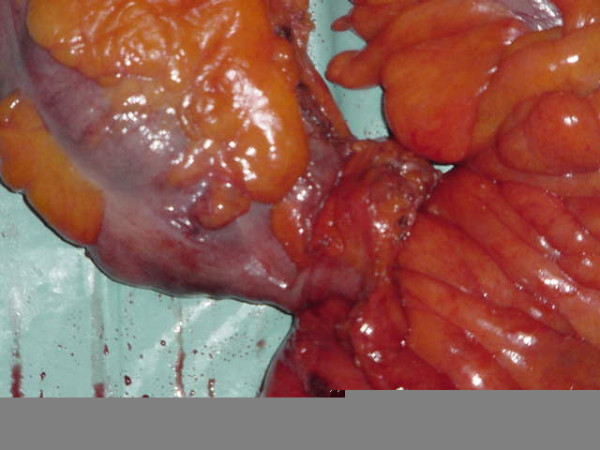
Appendiceal-sigmoid fistula

Our patient was treated with intravenous parenteral nutrition with adequate supplementation of intravenous fluids, correction of electrolytes and an antibiotic supportive treatment. As his condition worsened, he underwent surgery. An explorative laparotomy revealed that the entire colon was distended and pus was found around an appendiceal-sigmoid fistula (Figure 2). A proctocolectomy with ileostomy was performed and our patient was discharged without complications 12 days after surgery.

Three months after surgery, an ileal pouch-anal anastomosis was performed. Subsequently, our patient has remained in good general health.

## Discussion

UC is a chronic disease characterized by diffuse mucosal inflammation limited to the colon. Once diagnosis is confirmed, the anatomic extent of damage is assessed endoscopically. Limited colitis refers to disease limited to the colon, distal to the splenic flexure; pathology extending proximal to the splenic flexure is termed extensive colitis [1].

UC remains a disease found most commonly in teenagers and young adults, with a peak incidence between 20 and 29 years of age [2]. However, a second peak incidence has also been reported between 60 and 70 years of age in many studies [2].

A hospital in the United Kingdom serving a population of 300,000 would typically see 45 to 90 new cases of IBD each year [3,4]. UC affects approximately 250,000 to 500,000 individuals in the United States of America with an incidence of two to seven per 100,000 each year [1].

Therapy for UC is a rapidly evolving field, with many new biological agents under investigation that are likely to change therapeutic strategies radically in the next decade [3]. Surgical management of UC depends on the indications for surgery, the surgical setting (elective versus emergency), anal sphincter function, and the overall health and age of the patient [3,4]. Indications for surgery are intractability (49%), stricture, dysplasia, toxic colitis, hemorrhage and perforation [2].

We report an unusual complication of UC - an appendiceal-sigmoid fistula seen at colonoscopy (Figure 1). Our patient was referred to us with a toxic megacolon. After four days of medical therapy (intravenous fluids, parenteral nutrition, correction of electrolytes and antibiotic supportive treatment), our patient underwent surgery due of a worsening of the disease. During explorative laparotomy, we found that the whole colon was distended and pus was present around an appendiceal-sigmoid fistula. A proctocolectomy with ileostomy was performed. Our patient did not develop any complications and was discharged 12 days following surgery.

We believe that an inflamed appendix had perforated into our patient's sigmoid colon, producing a fistula and causing toxic colitis.

Three months after surgery, an ileal pouch-anal anastomosis was performed and subsequently our patient has remained in good general health. To the best of our knowledge, no cases of appendiceal-sigmoid fistula in patients affected by UC have been described in the literature; fistulae between the appendix and sigmoid tract are rarely reported for cases of diverticular disease [5-7] and appendicitis [8].

## Conclusions

UC is a chronic disease of the colon with significant incidences [1,2]. This unusual case highlights a patient with an appendiceal-sigmoid fistula complicated by UC in toxic colitis. To the best of our knowledge, this is the first such case reported in the literature.

## Abbreviations

CT: computed tomography; IBD: inflammatory bowel disease; UC: ulcerative colitis.

## Competing interests

The authors declare that they have no competing interests.

## Authors' contributions

MS was responsible for the conception and design of the study and translation of the article. RL was responsible for drafting the article. MD was responsible for the bibliography, photos and provision of study materials. SAC and AR collected and assembled the data. VM was responsible for logistic support and drafting the article. All authors read and approved the final manuscript.

## Consent

Written informed consent was obtained from the patient for the publication of this case report and any accompanying images. A copy of the written consent is available for review by the Editor-in-Chief of this journal.
